# Supplementary material for: FlywheelTools: Data Curation and Manipulation on the Flywheel Platform
Source: Front Neuroinform. 2021 Jun 22;15:678403. doi: 10.3389/fninf.2021.678403 (PMC8258420; doi:10.3389/fninf.2021.678403)
Supplement: Supplementary file 1 [file Table_1.DOCX]

def create_key(template, outtype=('nii.gz',), annotation_classes=None):

if template is None or not template:

raise ValueError('Template must be a valid format string')

return template, outtype, annotation_classes

# Create Keys

t1w = create_key(

'sub-{subject}/ses-{session}/anat/sub-{subject}_ses-{session}_T1w')

# loop over the seqinfo table

def infotodict(seqinfo):

# the dictionary of keys and list of files they correspond to

info = {

t1w: []

}

# loop over each row of your seqinfo table

for s in seqinfo:

print(s)

# if the series description contains "MPRAGE",

# add the DICOM identifier to the dictionary

if "MPRAGE" in s.series_description:

info[t1w].append(s.series_id)

return info
